# A regional modification to the Revised Swiss System for clinical staging of hypothermia including confusion

**DOI:** 10.1186/s13049-024-01273-3

**Published:** 2024-11-11

**Authors:** Duncan Gray, Mathieu Pasquier, Hermann Brugger, Martin Musi, Peter Paal

**Affiliations:** 1 Department of Emergency Medicine, Raigmore Hospital, Old Perth Road, Inverness, IV2 3UJ UK; 2 International Commission for Mountain Emergency Medicine (ICAR MedCom), Zürich, Switzerland; 3 Department of Emergency Medicine, Lausanne University Hospital, Lausanne, Switzerland; 4 Institute of Mountain Emergency Medicine, Eurac Research, Bolzano, Italy; 5 Department of Emergency Medicine, University of Colorado, Anschutz Medical Campus, Mail Stop B-215, 12401 17th Avenue, Aurora, CO 800045 USA; 6 Department of Anaesthesiology and Intensive Care Medicine, Hospitallers Brothers Hospital, Paracelsus Medical University, Salzburg, Austria

The Revised Swiss System (RSS) [1], uses conscious level as the primary element for field staging of accidental hypothermia, using the AVPU scale (Alert, Verbal, Pain and Unresponsive). The stages achieved estimate the risk of hypothermic cardiac arrest (HCA) rather than estimated core temperature (CT). After the publication of the RSS, Barrow et al. [2] conducted a retrospective study of the relationship between conscious level and the risk of HCA. They similarly used AVPU, but then divided the “Alert” group into “Alert confused” and “Alert not confused”. They found that those in their confused group had a risk of HCA (3 out of 12 patients) while the alert group had no risk of HCA (0 out of 33 patients). Despite the low numbers of patients in the confused group, this new finding reached statistical significance (p = 0.016). Within the context of their study, Barrow et al. [2] concluded that “any change in cognition proved to carry a significant risk of cardiac arrest”.

The RSS, using the AVPU scale, does not reflect this new finding, however it can be accommodated by using a variation of the AVPU scale which is in use: the ACVPU scale, where C is new confusion. ACVPU was originally conceived as part of the British National Early Warning Score (NEWS) 2 system in 2017 [3]. NEWS 2 is now the standard early warning system in United Kingdom (UK) hospitals and ambulance services and is increasingly used internationally [3]. ACVPU is used by the Resuscitation Council UK, in Advance Life Support and related courses and in guidelines [4], and throughout UK mountain rescue [5]. The “C” of ACVPU is defined as new confusion and includes disorientation or any new alteration to mentation and may be subtle. This definition of confusion is very similar to that used by Barrow et al. [2]. ACVPU therefore fits well as an alternative staging framework for accidental hypothermia.

We therefore propose a regional modification to the RSS, using the ACVPU scale (Fig. 1), for use in areas where ACVPU is the norm, such as the UK. Using ACVPU, in addition to incorporating the findings by Barrow et al. [2] regarding confusion, reflects the continuum of conscious level and HCA risk.

**Fig. 1:**
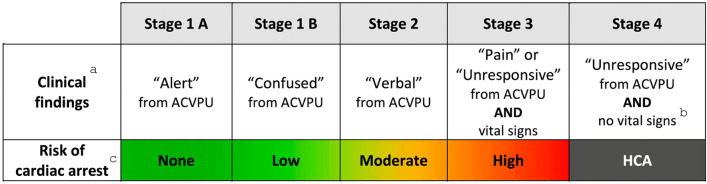
**a** In this regional modification to the Revised Swiss System, “Alert” corresponds to a GCS score of 15; “Confused” corresponds to a GCS score of 14, “Verbal” corresponds to a GCS score of 9–13; “Pain” and “Unresponsive” correspond to a GCS score < 9. While shivering is not used as a stage-defining sign in this regional modification to the Revised Swiss System, its presence usually means that the temperature is > 30 °C, a temperature at which hypothermic CA is unlikely to occur [6]. **b** No respiration, no palpable carotid or femoral pulse, no measurable blood pressure. Check for signs of life (pulse and, especially, respiration) for up to 1 min [7].** c** The transition of colours between stages represents the overlap of patients within groups. The estimated risk of cardiac arrest is based on accidental hypothermia being the only cause of the clinical findings. If other conditions impair consciousness, such as asphyxia, intoxication, high altitude cerebral oedema or trauma, this regional modification to the Revised Swiss System may falsely predict a higher risk of cardiac arrest due to hypothermia. Caution should be taken if a patient remains “alert”, “confused” or “verbal” while showing signs of haemodynamic or respiratory instability such as bradycardia, bradypnoea, or hypotension because this may suggest transition to a stage with higher risk of cardiac arrest

The main concepts of the RSS, using conscious level as the primary element to achieve staging and the stages being defined using the risk of HCA rather than core temperature ranges, were reaffirmed in the study by Barrow et al. [2] They found that the risk of HCA increased directly with impairment of conscious level and even suggest that consciousness alone may be at least as good as CT in predicting HCA risk. This regional modification builds on the formative RSS and continues the evolution of the Swiss System, by using ACVPU to provide more refinement of staging of mild hypothermia for use in areas where ACVPU is used.

## Data Availability

No datasets were generated or analysed during the current study.

## Abbreviations

RSS: Revised Swiss System
AVPU: Alert, verbal, pain and unresponsive
HCA: Hypothermic cardiac arrest
CT: Core temperature
ACVPU: Alert, confused, verbal, pain and unresponsive
NEWS: National Early Warning Score
UK: United Kingdom
