# Correction: A regional modification to the Revised Swiss System for clinical staging of hypothermia including confusion

**DOI:** 10.1186/s13049-024-01303-0

**Published:** 2025-01-03

**Authors:** Duncan Gray, Mathieu Pasquier, Hermann Brugger, Martin Musi, Peter Paal

**Affiliations:** 1 Department of Emergency Medicine, Raigmore Hospital, Old Perth Road, Inverness, IV2 3UJ UK; 2 International Commission for Mountain Emergency Medicine (ICAR MedCom), Zürich, Switzerland; 3 Department of Emergency Medicine, Lausanne University Hospital, Lausanne, Switzerland; 4 Institute of Mountain Emergency Medicine, Eurac Research, Bolzano, Italy; 5 Department of Emergency Medicine, University of Colorado, Anschutz Medical Campus, Mail Stop B-215, 12401 17th Avenue, Aurora, CO 800045 USA; 6 Department of Anaesthesiology and Intensive Care Medicine, Hospitallers Brothers Hospital, Paracelsus Medical University, Salzburg, Austria

**Correction: Scand J Trauma Resusc Emerg Med (2024) 32:110** 10.1186/s13049-024-01273-3

Following the publication of the original article, the authors reported that Fig. 1 was incomplete, and the legend was missing. The authors have provided the correct figure and legend, as well as updated the reference list. Two additional references cited in Fig. 1 are:

[6] Pasquier M, Hugli O, Paal P, Darocha T, Blancher M, Husby P, et al. Hypothermia outcome prediction after extracorporeal life support for hypothermic cardiac arrest patients: the HOPE score Resuscitation. 2018;126:58–64

[7] Lott C, Truhlar A, Alfonzo A, Barelli A, Gonzalez-Salvado V, Hinkelbein J, et al. European Resuscitation Council Guidelines 2021: Cardiac arrest in special circumstances Resuscitation. 2021;161:152–219

**Incorrect figure:**

**Figure:**
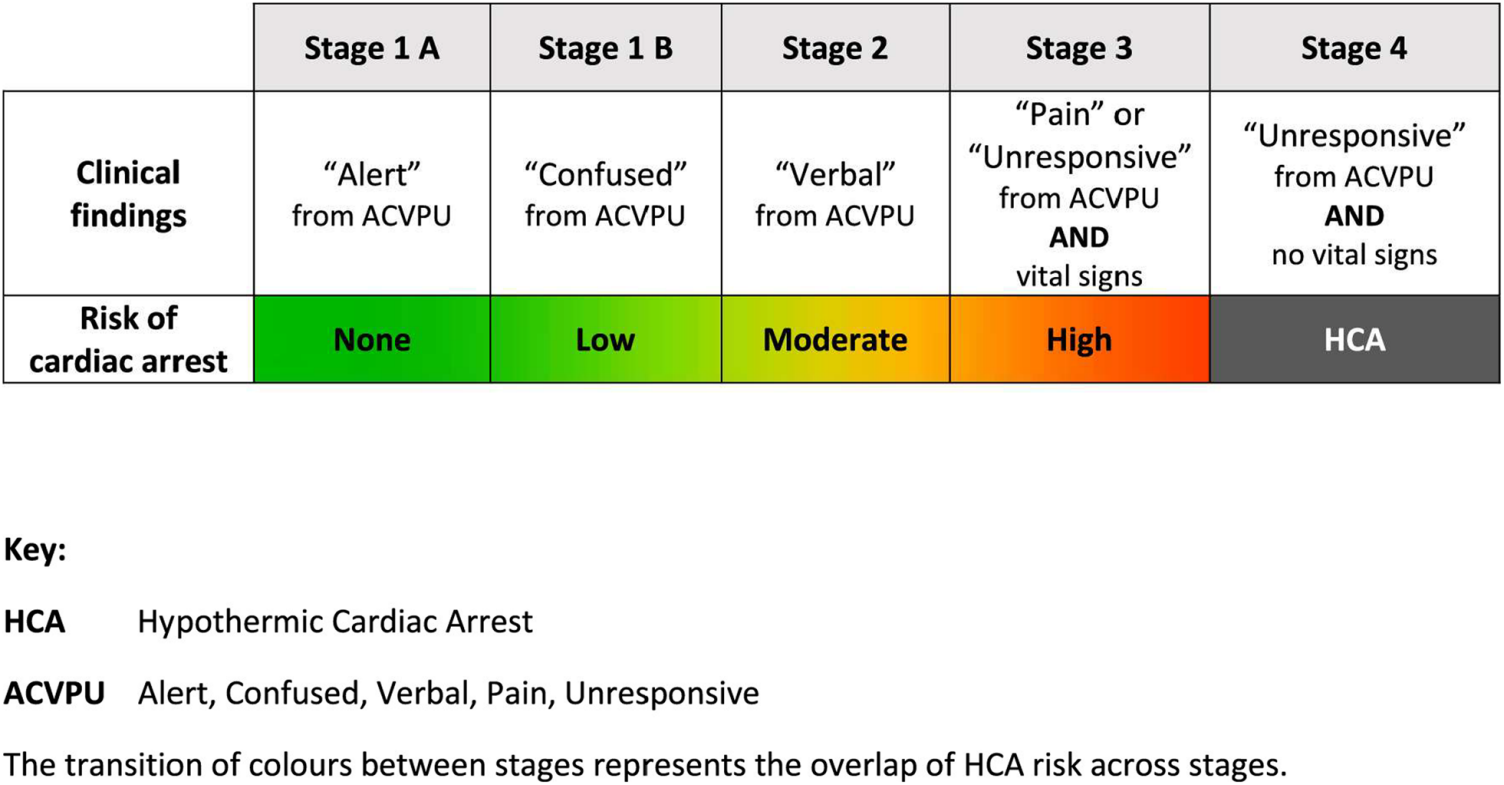

**Correct figure:**

**Figure:**
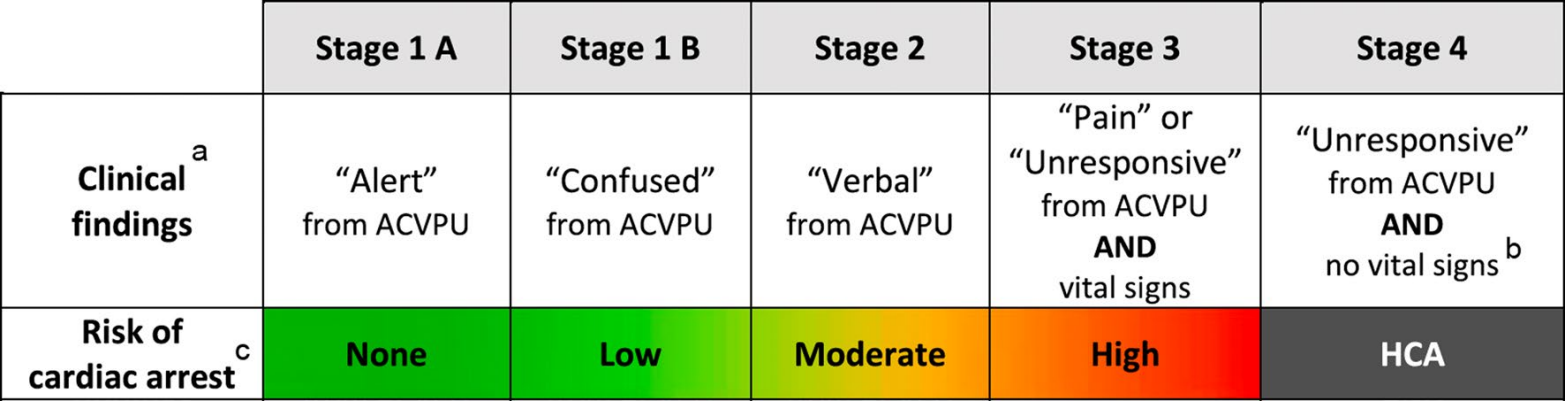

^a^In this regional modification to the Revised Swiss System, “Alert” corresponds to a GCS score of 15; “Confused” corresponds to a GCS score of 14, “Verbal” corresponds to a GCS score of 9–13; “Pain” and “Unresponsive” correspond to a GCS score < 9. While shivering is not used as a stage-defining sign in this regional modification to the Revised Swiss System, its presence usually means that the temperature is > 30 °C, a temperature at which hypothermic CA is unlikely to occur [6].

^b^No respiration, no palpable carotid or femoral pulse, no measurable blood pressure. Check for signs of life (pulse and, especially, respiration) for up to 1 min [7].

^c^The transition of colours between stages represents the overlap of patients within groups. The estimated risk of cardiac arrest is based on accidental hypothermia being the only cause of the clinical findings. If other conditions impair consciousness, such as asphyxia, intoxication, high altitude cerebral oedema or trauma, this regional modification to the Revised Swiss System may falsely predict a higher risk of cardiac arrest due to hypothermia. Caution should be taken if a patient remains “alert”, “confused” or “verbal” while showing signs of haemodynamic or respiratory instability such as bradycardia, bradypnoea, or hypotension because this may suggest transition to a stage with higher risk of cardiac arrest.

The reference list has been updated.

The Original Article has been corrected.

